# Impaired Iron-Copper Metabolic Axis in Human Intestinal Mucosa After Roux-en-Y Gastric Bypass: A Prospective Transcriptomic Study Using Double-Balloon Enteroscopy

**DOI:** 10.1007/s11695-026-08713-2

**Published:** 2026-05-09

**Authors:** Camila Shimizu, Dan Linetzky Waitzberg, Raquel Susana Torrinhas, Steven B. Heymsfield, Mariana Doce Passadore, Priscila Sala

**Affiliations:** 1 Curso de Nutrição, Centro Universitário São Camilo, São Paulo, Brazil; 2 Laboratório de Nutrição e Cirurgia Metabólica (LIM35), Faculdade de Medicina da Universidade de São Paulo, São Paulo, Brazil; 3 Pennington Biomedical Research Center, Louisiana, USA

## Abstract

**Background:**

Roux-en-Y gastric bypass (RYGB) effectively leads to weight loss in patients with severe obesity but is often accompanied by refractory iron deficiency. The molecular mechanisms governing the human intestinal copper-iron metabolic axis post-surgery remain poorly defined. This study evaluated the longitudinal impact of RYGB on intestinal gene expression related to micronutrient homeostasis.

**Methods:**

In a prospective study of 20 women (body mass index, BMI 46.5 ± 5.3 kg/m²), intestinal mucosa biopsies were collected via double-balloon enteroscopy (DBE) pre- and three months post-RYGB from anatomically marked sites in the duodenum, jejunum, and ileum. Gene expression was assessed using Affymetrix microarrays, with Ceruloplasmin (CP) validated by RT-qPCR.

**Results:**

Three months post-RYGB, BMI significantly (*p* < 0.05) decreased to 38.2 ± 4.2 kg/m². Microarray analysis revealed segment-specific adaptations. Divalent metal transporter 1 (DMT1) was significantly (*p* < 0.05) upregulated in the bypassed duodenum (+ 0.421), indicating a local compensatory response to decreased iron availability. Conversely, CP expression was significantly downregulated across all segments, notably in the jejunum (− 1.017; *p* < 0.05). This downregulation, confirmed by RT-qPCR, suggests impaired iron oxidation and mobilization. Lysyl oxidase (LOX), a copper-dependent enzyme critical for mucosal structural integrity, was also significantly downregulated in the duodenum (− 0.333; *p* < 0.05) and jejunum (− 0.450; *p* < 0.05).

**Conclusion:**

RYGB induces early and segment-specific transcriptional adaptations in the intestinal mucosa. The upregulation of DMT1 in the bypassed duodenum suggests a compensatory response, whereas the consistent downregulation of CP and LOX indicates a potential alteration in the copper–iron metabolic axis. These findings provide a biologically plausible framework for postoperative micronutrient disturbances; however, in the absence of functional and biochemical data, causal relationships cannot be established.

## Key Points

Longitudinal Molecular Mapping: Roux-en-Y gastric bypass (RYGB) triggers rapid and segment-specific transcriptional shifts in the human intestinal mucosa within three months postoperatively.

The Copper-Iron Bottleneck: Systemic downregulation of Ceruloplasmin (CP) and Lysyl Oxidase (LOX) suggests that postoperative iron deficiency is exacerbated by impaired copper-dependent iron mobilization and transport.

Active Mucosal Adaptation: Significant upregulation of DMT1 in the bypassed duodenum indicates a localized, compensatory effort to enhance iron uptake, despite the absence of direct nutrient transit.

Clinical Implications: These findings provide a mechanistic basis for refractory anemia and underscore the necessity for integrated, early monitoring of the copper-iron metabolic axis to optimize nutritional outcomes.

## Introduction

### Obesity as a Global Challenge

Obesity is a chronic disease characterized by excessive fat accumulation and serves as a primary risk factor for cardiovascular diseases, type 2 diabetes mellitus (T2DM), and various malignancies [1–3]. While bariatric surgery, specifically Roux-en-Y gastric bypass (RYGB), remains the most effective intervention for sustained weight loss and metabolic improvement [4–6], it imposes significant challenges on micronutrient homeostasis.

### The Nutritional Cost of Anatomical Alteration

RYGB involves profound anatomical reconstruction, excluding the distal stomach, duodenum, and proximal jejunum from the nutrient stream [7–9]. Although this reconfiguration promotes satiety and weight loss, it bypasses the primary sites of mineral absorption. Consequently, 65% to 80% of patients experience micronutrient deficiencies postoperatively, with copper (Cu) and iron deficiencies being among the most clinically significant and refractory [4, 8, 10, 11].

### The Iron-Copper Paradox

Recent data indicate that copper deficiency prevalence can reach 28% within two years post-RYGB, potentially leading to severe myeloneuropathy [12–14]. Similarly, more than 20% of patients develop iron deficiency anemia within five years [15, 16]. Critically, the deficiencies of these two minerals are not independent; their metabolisms are biochemically intertwined through copper-dependent enzymes. Intestinal absorption of iron is mediated by proteins such as divalent metal transporter 1 (DMT1), while its systemic mobilization requires the multi-copper ferroxidase ceruloplasmin (CP). CP is essential for oxidizing ferrous iron (Fe²⁺) into ferric iron (Fe³⁺), the only form capable of binding to transferrin for transport [17, 18]. Furthermore, copper is a vital cofactor for lysyl oxidase (LOX), an enzyme necessary for maintaining the structural integrity of the intestinal mucosa.

### The Knowledge Gap and Study Objective

While the clinical manifestations of these deficiencies are well-documented, the early transcriptional adaptations of the human intestinal mucosa following RYGB remain largely unexplored. Our group has previously demonstrated that RYGB triggers significant shifts in genes related to B12, folate, and vitamin A absorption [19–21]. However, it remains unclear how the bypassed and alimentary segments molecularly adapt to the altered mineral flux.

This study aims to evaluate longitudinal changes in the expression of key genes involved in the copper-iron metabolic axis—specifically DMT1, CP, and LOX—using mucosal biopsies obtained via double-balloon enteroscopy (DBE). Understanding these early molecular adaptations is essential for transitioning from reactive to proactive nutritional management in the bariatric population.

## Materials and Methods

### Ethical Considerations

This prospective study was conducted in accordance with the ethical principles of the Declaration of Helsinki. The study protocol was approved by the local ethics committee. All participants provided written informed consent before study initiation.

### Patients and Surgical 

Procedures Between February 2011 and December 2014, 20 adult women (aged 18–60 years) scheduled for elective RYGB were enrolled. This study is nested within the multicentric SURMetaGIT, and specific inclusion/exclusion criteria have been previously reported [22]. To ensure cohort homogeneity, all patients underwent a standardized non-banded RYGB, creating a ~ 30 mL proximal gastric pouch with a 50–60 cm biliopancreatic limb and a 100–120 cm alimentary limb. This procedure effectively excludes the distal stomach, the entire duodenum, and the proximal jejunum from the nutrient stream. A project researcher supervised all surgeries to verify precise limb measurements and anastomotic consistency.

### Intestinal Biopsies Collection and Transcriptomic Analyses 

Double-balloon enteroscopy (DBE) was performed at the Gastrointestinal Endoscopy Service of HC-FMUSP approximately two weeks before and three months after RYGB. The procedure was conducted using an anterograde (oral) approach by an experienced endoscopy team, enabling reliable access to proximal small bowel segments, including the duodenum and jejunum, through a push-and-pull technique that allows deep intubation and stabilization for tissue sampling. Patients fasted for 12 h and discontinued oral medications and supplements (except antihypertensives) for 3 to 5 days prior to the procedure to minimize the potential effects of these substances on intestinal gene expression. During the procedure, mucosal biopsies (approximately 15–20 mg per segment) were obtained from the duodenum, jejunum, and ileum and were immediately stored in liquid nitrogen. In the preoperative examination, biopsy sites were marked using SPOT^®^ ink, allowing for sample collection from anatomically similar locations during the postoperative evaluation. This approach enabled paired longitudinal sampling of intestinal mucosa across all targeted segments. Total RNA was extracted and purified from the collected samples using the RNeasy Plus Mini Kit (Qiagen™, USA), following the manufacturer’s instructions. Gene expression was assessed through microarray analysis using the Human Gene Chip 1.0 ST Array, according to the manufacturer’s guidelines, as part of a previously completed project. Genes involved in iron and copper metabolic pathways were selected for evaluation and interpretation in the microarray analyses [23–25]. Gene expression data were submitted to statistical analysis, using fold change (FC) to quantify the magnitude and direction of gene expression changes. An FC of 0 indicates no change, while positive and negative values correspond to an increase and decrease, respectively. The CP gene was validated using quantitative RT-PCR with TaqMan gene expression assays (Life Technologies, Carlsbad, CA, USA), following the manufacturer’s recommendations [26, 27]. The detailed methodology, including the CONSORT diagram of biopsy sampling and RNA quality for microarray analysis, has been previously described in the SURMetaGIT study [22] and in our prior publication [19].

## Results

### Patient Clinical Outcomes

The baseline and 3-month post-RYGB clinical characteristics of the participants have been previously published [19]. The mean age of the cohort was years. Using each patient as her own control, we observed that three months after the procedure, all 20 patients showed a significant reduction in body weight from 115.0 ± 16.0 kg to 94.5 ± 12.8 kg (*P* < 0.05). Correspondingly, the mean body mass index (BMI) decreased from baseline to three months post-RYGB.

### Transcriptomic Profiling and Mucosal Adaptation

The use of DBE allowed for the successful collection of high-quality tissue across all targeted segments. Microarray analysis revealed that RYGB triggered early and segment-specific transcriptional shifts in genes associated with iron and copper homeostasis, suggesting rapid intestinal adaptations in the postoperative period.

**Fig. 1:**
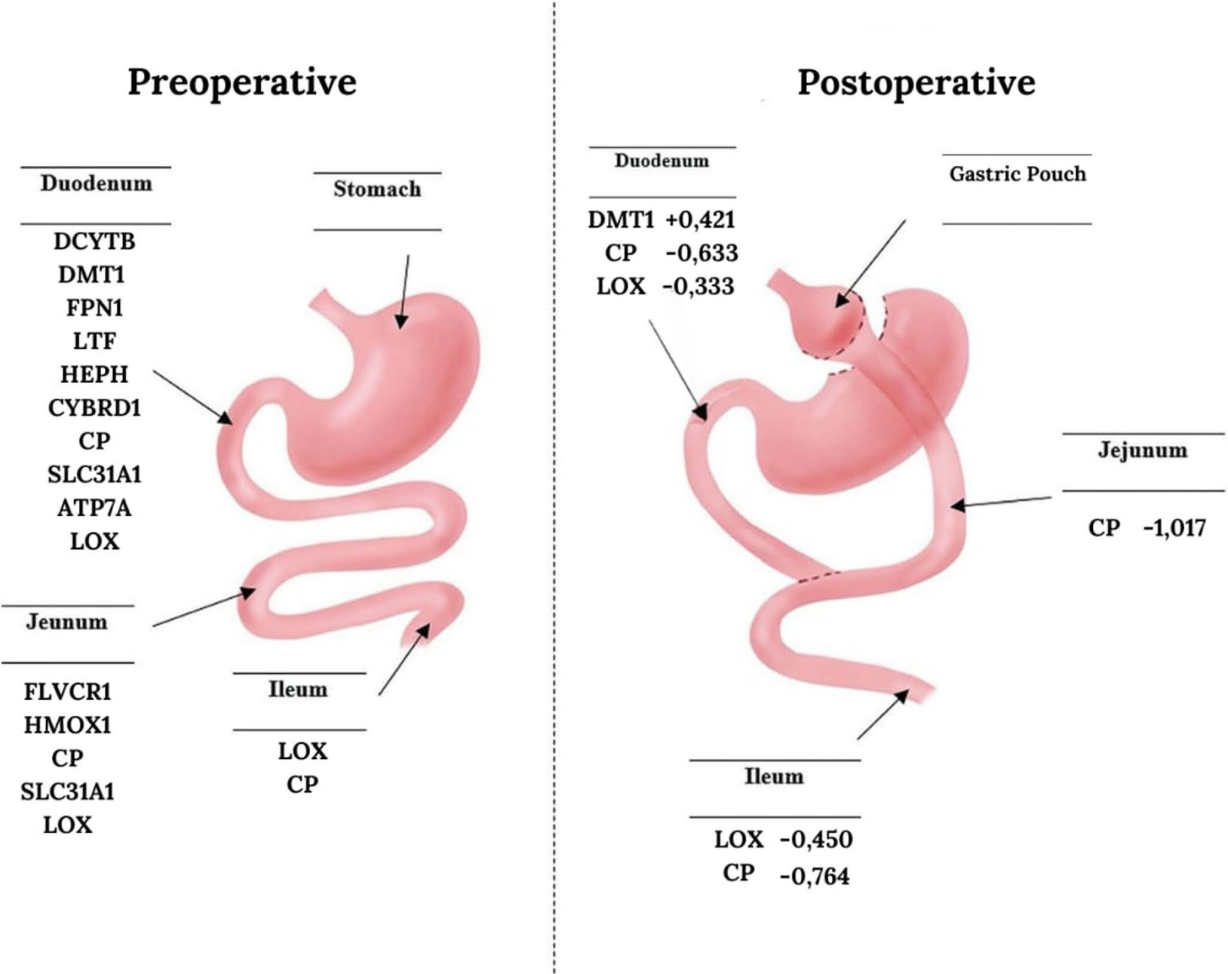
**Variation in gene expression from microarray analysis before and after RYGB, indicating its main sites of expression**. Schematic representation of intestinal segments and gene expression changes related to iron and copper metabolism before and three months after Roux-en-Y gastric bypass (RYGB). In the preoperative state, genes involved in iron and copper transport and homeostasis are expressed along the duodenum, jejunum, and ileum. Postoperatively, significant transcriptional alterations were observed, including upregulation of DMT1 in the bypassed duodenum and downregulation of copper-dependent genes such as CP and LOX across intestinal segments, particularly in the jejunum and ileum. Values represent fold changes in gene expression relative to the preoperative condition

Before the procedure, it was verified that the genes were expressed at constitutive levels appropriate for mineral absorption. After RYGB, with altered gastrointestinal anatomy, microarray analysis showed significant changes in the expression of these genes (Fig. 1).

### Iron and Copper Homeostasis Pathways

Gene expression profiling indicated a complex biological response to the surgical exclusion of the proximal intestine. An increase in DMT1 expression was noted specifically in the bypassed duodenum (+ 0.421, *p* < 0.05), which likely represents a localized compensatory effort to optimize iron uptake in the absence of nutrient transit.

In contrast, ceruloplasmin (CP) showed a significant and systematic reduction in all intestinal regions. The downregulation was most acute in the jejunum (−1.017, *p* < 0.05), followed by the ileum (−0.764, *p* < 0.05) and the bypassed duodenum (−0.633, *p* < 0.05). This widespread suppression of CP expression, which was independently validated by RT-qPCR, suggests a potential systemic failure in the copper-dependent oxidation and transport of iron. Additionally, the LOX gene showed lower expression in the duodenum (−0.333, *p* < 0.05) and jejunum (−0.450, *p* < 0.05). This downregulation of a key copper-dependent enzyme may signify a compromise in mucosal structural integrity and extracellular matrix remodeling post-RYGB (Table 1).

**Table 1:** Segment-specific gene expression changes of CP, DMT1, and LOX following RYGB, assessed by microarray and RT-qPCR

Gene	Intestinal Segment	Microarray (FC)	RT-qPCR (FC)
CP	Duodenum	−0,633*	−0,545
	Jejunum	−1,017*	−0,216*
	Ileum	−0,764*	−0,556
DMT1	Duodenum	+ 0,421*	-----------
	Jejunum	−0,070	-----------
	Ileum	+ 0,130	-----------
LOX	Duodenum	−0,333	-----------
	Jejunum	−0,450	-----------
	Ileum	−0,184	-----------

Values are expressed as fold change (FC). Negative and positive values indicate decreased and increased gene expression, respectively. Statistically significant changes (P ≤ 0.05) are indicated by (*)

Microarray analyses were performed in intestinal biopsies with adequate RNA quality, as previously reported [19], with sample sizes varying by segment: duodenum (n = 14), jejunum (n = 16), and ileum (n = 12)

A larger sample size was included for RT-qPCR validation, comprising the original cohort plus additional patients, resulting in the following sample sizes: duodenum (n = 17), jejunum (n = 20), and ileum (n = 18) [19]

RT-qPCR validation was performed only for CP, whereas DMT1 and LOX expression levels are based exclusively on microarray data and were not independently validated

## Discussion

The anatomical alterations of the gastrointestinal tract following RYGB are primary drivers of the metabolic and nutritional disturbances observed in postoperative patients [19]. Our longitudinal analysis using paired intestinal biopsies revealed early and segment-specific transcriptional adaptations in genes involved in iron and copper homeostasis, providing novel insights into the molecular mechanisms underlying postoperative micronutrient deficiencies.

Notably, we observed a significant upregulation of DMT1 in the bypassed duodenum. This indicates that this excluded segment remains metabolically active and capable of mounting a localized adaptive response to altered systemic mineral availability, even in the absence of direct nutrient exposure This finding is consistent with prior studies [28, 29] suggesting that the bypassed limb contributes to systemic mineral sensing.

In contrast, ceruloplasmin (CP), a multi-copper ferroxidase essential for the conversion of ferrous iron (Fe²⁺) to ferric iron (Fe³⁺) prior to transferrin binding, exhibited a marked and systemic reduction in expression across all intestinal segments. This downregulation was most pronounced in the jejunum, a primary site for mineral absorption post-RYGB. The RT-qPCR validation confirmed the robust and consistent nature of these changes. Functionally, reduced CP expression may represent a bottleneck in iron metabolism, whereby intracellular iron uptake may occur, but its subsequent oxidation and release into the circulation are compromised.

Copper serves as an indispensable cofactor for iron metabolism. The copper-iron axis is regulated by transporters like Ctr1 and ATP7A, which facilitate the incorporation of copper into cuproenzymes such as CP and LOX [30–33]. In our study, the LOX gene, responsible for cross-linking collagen and elastin, was significantly downregulated in the duodenum and jejunum. This suppression of LOX may signal a previously unrecognized risk of compromised mucosal structural integrity and impaired extracellular matrix remodeling following surgery [32, 34, 35].

The interplay between iron and copper metabolism is well-established. A deficit in CP not only impairs intestinal iron flux but may also hinder iron release from hepatic stores, potentially explaining why some patients remain anemic despite oral iron supplementation [28, 33, 35, 36]. Furthermore, because DMT1 can transport both metals, its upregulation in iron-deficient states could paradoxically lead to excessive copper uptake in specific contexts, predisposing patients to hepatic copper imbalances [28, 29].

This raises the possibility that disruptions in the copper–iron axis may be qualitative—at the transcriptional or functional level—rather than purely quantitative, potentially contributing to the variability in clinical responses observed after RYGB.

Importantly, our findings demonstrate that these molecular disruptions manifest as early as three months postoperatively. Although clinically overt deficiencies are typically reported later [33], these findings suggest that early transcriptional changes may precede subsequent biochemical and clinical manifestations. This reinforces the need for early and comprehensive nutritional monitoring rather than waiting for symptomatic deficiency to appear. Copper supplementation after RYGB should be interpreted within the framework of established postoperative nutritional guidelines rather than as a targeted strategy to reverse transcriptional alterations. Routine preventive supplementation (e.g., ~ 2 mg/day, as recommended by the American Society for Metabolic and Bariatric Surgery) is intended to maintain adequate systemic copper status in this high-risk population. However, the present findings do not support the assumption that increased copper intake would directly restore the expression of copper-dependent genes such as CP or LOX [37].

A key limitation of this study is the absence of concomitant biochemical and clinical data, including serum copper, ceruloplasmin, and iron parameters, which precludes direct correlation between intestinal gene expression and systemic micronutrient status. This reflects the original design of the SURMetaGIT study, which was primarily focused on metabolic outcomes rather than micronutrient metabolism. Therefore, the present results should be interpreted as a secondary, exploratory analysis based on transcriptomic data.

In addition, the relatively small sample size and short follow-up period limit the generalizability and temporal interpretation of the findings. However, the use of a longitudinal paired design, in which each participant served as her own control, reduces inter-individual variability and strengthens the detection of biologically relevant transcriptional changes. The inclusion of only female participants, while reducing biological variability and enhancing internal consistency, may further limit generalizability to male populations.

Potential confounding effects of prior medication use should also be acknowledged. Although a washout period was implemented before tissue collection, residual or cumulative effects of commonly used medications cannot be entirely excluded. Nevertheless, the segment-specific and biologically coherent gene expression patterns observed suggest that these findings are unlikely to be explained solely by nonspecific pharmacological effects.

From a methodological perspective, the use of microarray technology represents an additional limitation, as it provides lower resolution compared to next-generation sequencing approaches. Future studies employing RNA sequencing may enable a more comprehensive characterization of intestinal molecular adaptations following RYGB.

Finally, although this study focuses on early molecular adaptations, it is embedded within a broader prospective research platform with ongoing clinical follow-up. Future studies integrating transcriptomic, biochemical, and clinical data will be essential to determine the functional significance and long-term clinical impact of these findings.

## Conclusion

In summary, RYGB induces early and segment-specific transcriptional adaptations in the human intestinal mucosa. The upregulation of DMT1 in the bypassed duodenum suggests a compensatory response to altered iron availability, whereas the consistent downregulation of copper-dependent genes such as CP and LOX indicate a potential disruption of the copper–iron metabolic axis. These findings provide a biologically plausible framework that may contribute to postoperative micronutrient disturbances. However, in the absence of functional and biochemical data (e.g., circulating copper, ceruloplasmin activity, and iron parameters), causal relationships cannot be established. Therefore, the present results should be interpreted as early molecular signals that may precede clinically overt deficiencies. Future studies integrating transcriptomic, biochemical, and clinical outcomes are required to determine the functional relevance and long-term clinical implications of these alterations.

## Data Availability

No datasets were generated or analysed during the current study.
